# Intradialytic Changes and Prognostic Value of Ventriculo-Arterial Coupling in Patients With End-Stage Renal Disease: Protocol for an Observational Prospective Trial

**DOI:** 10.2196/71948

**Published:** 2025-06-23

**Authors:** Alessandro Salustri, Giovanni Tonti, Gianni Pedrizzetti, Aizhan Zhankorazova, Zaukiya Khamitova, Bauyrzhan Toktarbay, Dinara Jumadilova, Marina Khvan, Dinara Galiyeva, Makhabbat Bekbossynova, Murat Mukarov, Alexey Kokoshko, Abduzhappar Gaipov

**Affiliations:** 1 Nazarbayev University School of Medicine Astana Kazakhstan; 2 University of Chieti-Pescara Chieti Italy; 3 Department of Engineering and Architecture, University of Trieste Trieste Italy; 4 Heart Center University Medical Center Astana Kazakhstan; 5 Astana Medical University Astana Kazakhstan

**Keywords:** ventriculo-arterial coupling, pressure-volume loop, end-stage renal disease, hemodialysis, echocardiography

## Abstract

**Background:**

The acute effect of hemodialysis (HD) on left ventricular mechanics has been evaluated in several studies; however, their results are not consistent. Eventually, the heart and the arterial system behave as an interconnected system and not as isolated structures; thus, the evaluation of the interaction of cardiac contractility with the arterial system would provide a more comprehensive understanding of cardiovascular function and cardiac energetics. However, there have not been any studies demonstrating changes in terms of volumes, contractility, intraventricular pressure gradient distribution, and vascular properties in response to changes in loading conditions and their impact on the outcome in patients undergoing HD. Recently, a noninvasive method for assessing left ventricular pressure-volume loop and ventriculo-arterial coupling (VAC) from feature-tracking cardiac magnetic resonance or echocardiography has been proposed. We believe that this method allows a comprehensive evaluation of the hemodynamic status of the patients undergoing HD, including the relationships between cardiac function and arterial elastance, and might provide prognostic information.

**Objective:**

The primary objective of this study is to evaluate changes in VAC before and after a HD session. The secondary objective is to assess the prognostic value of VAC parameters in predicting adverse outcomes.

**Methods:**

A 2D transthoracic echocardiogram will be performed before and after a HD session in patients with end-stage renal disease. We target to enroll 323 patients. Images will be analyzed with advanced software based on speckle-tracking, able to reconstruct the pressure-volume loop. From the pressure-volume loop, arterial (Ea) and ventricular (Ees) elastance will be derived. VAC will be calculated as the Ea/Ees ratio. Patients will be followed up for 18 months. Primary endpoints will be a composite of all causes of death, nonfatal myocardial infarction, and hospitalization due to worsening heart failure.

**Results:**

The study received funding in August 2024, with patients’ enrollment scheduled to take place from January 1 to June 30, 2025. Data analysis will start in April 2025 and is expected to continue until June 2026. The findings of the study are tentatively planned for publication in the winter of 2027.

**Conclusions:**

This study will provide data on the changes in VAC induced by HD and their potential prognostic value. This assessment could be useful for tailoring volume depletion during HD and to improve patients’ outcomes.

**Trial Registration:**

ClinicalTrials.gov NCT06622928; https://clinicaltrials.gov/study/NCT06622928

**International Registered Report Identifier (IRRID):**

PRR1-10.2196/71948

## Introduction

### Background

One of the main goals of HD therapy is to remove from the patient the excess fluid that accumulates in the body due to kidney failure. Optimal fluid volume management is an essential component of HD adequacy, but the amplitude of volume fluctuation is still a quite challenging clinical condition. In fact, HD in patients with end-stage renal disease (ESRD) removes a large amount of fluid from the body in a few hours. Therefore, patients with ESRD on HD are characterized by central volume fluctuations related to volume expansion in the interdialytic period and fluid loss during HD. They also tend to have changes in cardiac structure and function, which have been recognized as key prognostic factors [1]. A myriad of mechanisms, such as chronic volume and pressure overload, anemia, uremia, high-flow arteriovenous shunts, fluid retention, abnormal calcium and phosphate metabolism, and hyperparathyroidism, can lead to cardiac abnormalities [2]. Furthermore, HD-induced acute hemodynamic changes in electrolytes, arterial pressure, blood volume, and sympathovagal balance can negatively impact cardiac function [3]. In fact, patients with chronic kidney disease exhibit a pronounced risk for cardiovascular events: 50% of all patients with chronic kidney disease stages 4 to 5 have cardiovascular diseases, and cardiovascular mortality accounts for approximately 40% to 50% of all deaths in patients with advanced chronic kidney disease (stage 4) as well as ESRD (stage 5), compared with 26% in controls with normal kidney function [4].

Changes in left ventricular (LV) volume during HD will alter LV contraction and strain. The acute effect of HD on echocardiographic indices of cardiac structure and function has been evaluated in several studies; however, their results are not uniform. Eventually, the heart and the arterial system behave as an interconnected system and not as isolated structures; thus, the evaluation of the interaction of cardiac contractility with the arterial system would provide a more comprehensive understanding of cardiovascular function and cardiac energetics. However, there is still scanty data on changes in volumes, contractility, intraventricular pressure gradient distribution, and vascular properties in response to changes in loading conditions and their impact on the outcome.

### Ventriculo-Arterial Coupling

The concept that the cardiovascular system works better when the heart and the arterial system are coupled has been well demonstrated [5-7]. When the heart pumps blood into the vascular tree at a rate and volume that matches the capability of the arterial system to receive it, both cardiovascular performance and its associated cardiac energetics are optimal [8]. A contractility or arterial tone that is too high or too low decouples these processes and can lead to cardiac failure independent of myocardial ischemia or the toxic effects of sepsis and related systemic disease processes. This optimization means that the LV workload and the arterial system optimally match when the LV ejects the blood into the arterial system and is quantified by ventriculo-arterial coupling (VAC) parameters. This process is optimized without excessive changes in LV pressure, and the mechanical energy of LV ejection is completely transferred from the ventricle to the arterial system [9].

VAC can be defined as the ratio of the arterial elastance (Ea) to the ventricular elastance (Ees). This ratio was first proposed by Suga and Sagawa [10] as a method to evaluate the mechanical efficiency of the cardiovascular system and the interaction between cardiac performance and vascular function. The Ea/Ees ratio has been consistently demonstrated to be a reliable and effective measure of cardiovascular performance [11]. The balance between myocardial oxygen consumption and the mechanical energy required to perform this work appears to be optimal when the heart and the peripheral vascular system are coupled (ie, the Ea/Es is near unity). In this case, the LV provides an adequate stroke volume with the lowest possible energetic consumption [12]. Thus, VAC is an effective index of the mechanical performance of the LV and the dynamic modulation of the cardiovascular system.

### Hypothesis and Study Aims

The relevance of VAC as a parameter of hemodynamic optimization could be related to the fact that VAC is a parameter of cardiovascular efficiency whereas the classical hemodynamic parameters are exclusively parameters of cardiovascular efficacy.

With these concepts in mind, the main scientific questions and hypotheses of this project are:

1. What is the impact of HD on ventricular function?
2. What is the impact of HD on ventricular and arterial elastance?
3. What is the prognostic value of intradialytic VAC changes?

Accordingly, we sought to study patients with ESRD to assess (1) VAC parameters before and after the HD session, and (2) the value of VAC parameters in predicting adverse outcomes.

## Methods

### Trial Design

This research is designed as an observational cohort study with a prospective approach. Participants will be enrolled and monitored over a period of time to assess how hemodynamic changes induced by HD influence future outcomes. As an observational study, the researchers will not intervene but will simply collect and analyze data as events occur naturally during the study period.

### Study Settings

The following study centers were used:

1. BBNura Center of Efferent Therapy and HD: the HD service has been operating in Astana since 1988. Four HD centers are working around the clock to provide emergency care to patients requiring renal replacement therapy.
2. Nazarbayev University is ranked in the top 30% of international research universities in the Times Higher Education World University Rankings 2024. The Doctor of Medicine program received full international accreditation from the Eurasian Centre for Accreditation and Quality Assurance in Higher Education and Health Care, recognized by the World Federation for Medical Education.
3. Heart Center-University Medical Center opened its doors on October 12, 2011. Today, it is the leading heart surgery clinic in Central Asia, offering tertiary medical and quality services.

### Recruitment

Patients with ESRD undergoing permanent HD in a specialized center in Astana, Kazakhstan, will be recruited (Textbox 1).

**Inclusion and exclusion criteria.**

**Inclusion criteria**

- Adults aged 18 years or older.
- Standard renal replacement schedule with 3 dialysis sessions per week.
- Dialysis vintage of at least 3 months.
- Dialysis adequacy with single-pool Kt/V >1.2 as recommended by international guidelines for thrice weekly HD.

**Exclusion criteria**

- Previous kidney transplantation.
- Myocardial infarction, unstable angina, or stroke during the previous 6 months.
- Severe stage III to IV congestive heart failure according to the New York Heart Association classification.
- Chronic atrial fibrillation or other known arrhythmia.
- History of nonadherence to the prescribed weekly dialysis schedule in the previous month.
- BMI >40 kg/m^2^.
- History of malignancy or other clinical conditions associated with very poor prognosis.
- The main reasons for these criteria were to avoid confounding factors that may affect the hemodynamic parameters and to exclude patients with unstable conditions.

### Expected Outcomes

Patients will be followed up for 18 months. Clinical variables and intradialytic changes in VAC parameters will be related to outcome events. The primary endpoint will be a composite of all causes of death, nonfatal myocardial infarction, and hospitalization due to worsening heart failure. Based on previous studies, we expect a mortality rate of around 10% per year [13], an incidence of acute myocardial infarction of 3.9 per 1000 patients per year [14], and an incidence of hospitalization due to worsening heart failure of 5.8 per 100 persons per year [15].

### Participant Timeline

The schedule of the study project including enrolment and assessment is outlined in Table 1.

**Table 1:** Schedule of the study project from 2024 to 2026.

Events		January	February	March	April	May	June	July	August	September	October	November	December
**2024**													
	IREC^a^ approval							✔					
	Hiring research assistants								✔				
	Logistic set-up (workstation, software, training with Medis imaging bioengineering)									✔	✔	✔	✔
	Checklist (VAC^b^)									✔	✔	✔	✔
	Manuscript preparation (review)								✔	✔	✔	✔	✔
**2025**													
	Patient recruitment, informed consent, and data collection from eligible participants	✔	✔	✔	✔	✔	✔	✔	✔	✔	✔	✔	✔
	Data analysis (VAC)				✔	✔	✔	✔	✔	✔	✔	✔	✔
	1-year follow-up			✔	✔	✔	✔	✔	✔	✔	✔	✔	✔
	Manuscript preparation: (VAC)							✔	✔	✔	✔	✔	✔
**2026**													
	Final data collection	✔	✔	✔									
	Data analysis (outcomes)	✔	✔	✔	✔	✔	✔						
	Manuscript preparation: (outcomes)							✔	✔	✔	✔	✔	✔

^a^IREC: Institutional Research Ethics Committee.

^b^VAC: ventriculo-arterial coupling.

### Sample Size

The sample size was calculated based on 95% CI, margin of error 5%, and estimated incidence of cardiovascular events 30%, as follows:



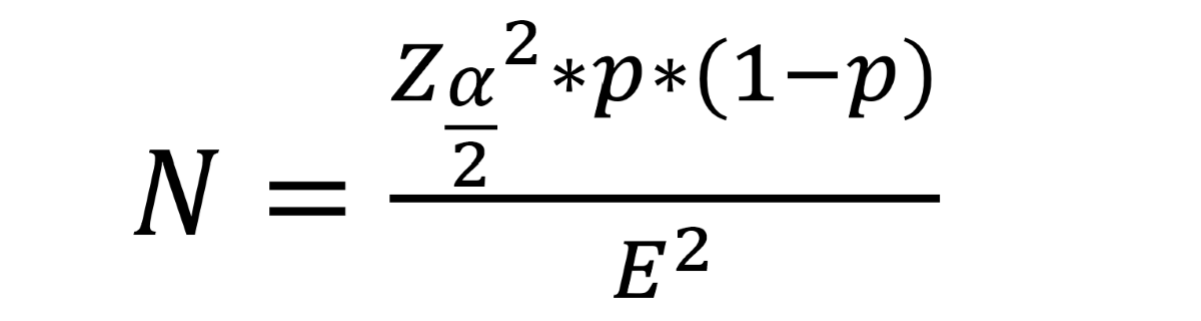



where N is the sample size, 
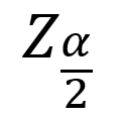
 is a critical value from the standard normal distribution corresponding to the desired 95% CI (1.96), *P* is the proportion of estimated incidence (0.3), and E is the desired margin of error (0.05) [16].

### Feasibility of Recruitment of the Sample Size

Patients on HD are often fragile and may not accept to undergo an additional investigation, although noninvasive. We will try to limit the number of noncompliant patients with several actions (Textbox 2).

**Action plan to reduce the number of uncompliant patients.**

- Involvement of the referring nephrologist in the explanation of the study.
- Clear explanation to the patient on the nature of a transthoracic echocardiogram (Multimedia Appendix 1).
- Patients are coming 3 times per week; thus, we will give them the freedom to choose any appropriate day for the study.
- We will provide a summary of the echocardiographic study to the dialysis center to be shared with the patients.

### Data Collection Methods

**Figure 1:**
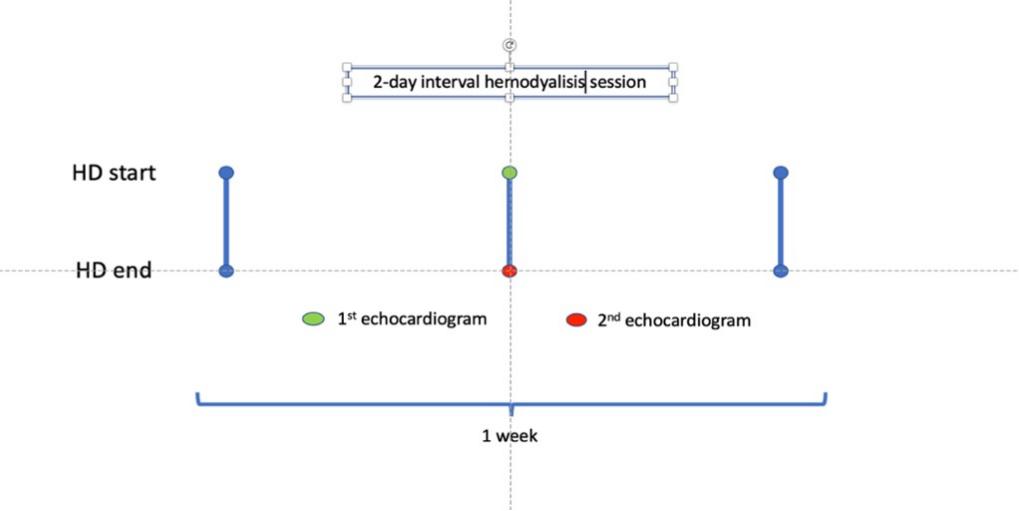
Time sequence of the echocardiography evaluation. HD: hemodialysis.

Demographic characteristics, full medical history, and dialysis-related parameters of study participants will be recorded on purpose-built data-collecting sheets (Multimedia Appendix 2). The study design diagram of the chronological sequence of the echocardiographic evaluations is presented in Figure 1. SPIRIT (Standard Protocol Items: Recommendations for Interventional Trial) reporting guidelines [17] have been used (Multimedia Appendix 3). Concomitant care and interventions during the HD session will be left to the attending nephrologist.

### Echocardiography

2D and Doppler echocardiogram will be acquired by a skilled sonographer before and immediately after the HD session. Care will be taken in acquiring 2D apical views (4-chamber, 2-chamber, and long-axis views) aiming for the best visualization of the LV endocardial border. Images will be saved on digital media storage devices and analyzed offline at the echocardiographic core laboratory of our institution.

Echocardiographic images will be analyzed offline using dedicated software based on a mathematical model [18] (QStrain, Medis BV). The LV end-diastolic and end-systolic volumes and ejection fraction will be determined from the modified biplane Simpson method. Left atrial volume will be calculated using the biplane area-length method. LV volume and mass and left atrial volume will be subsequently indexed by body surface area. The peak early filling (E wave), the late diastolic (A wave) velocities, and the deceleration time will be assessed from the transmitral flow. The peak systolic (s’), early diastolic (e’), and late diastolic (a’) mitral annular velocities will be measured at the septal annulus using spectral Doppler.

**Figure 2:**
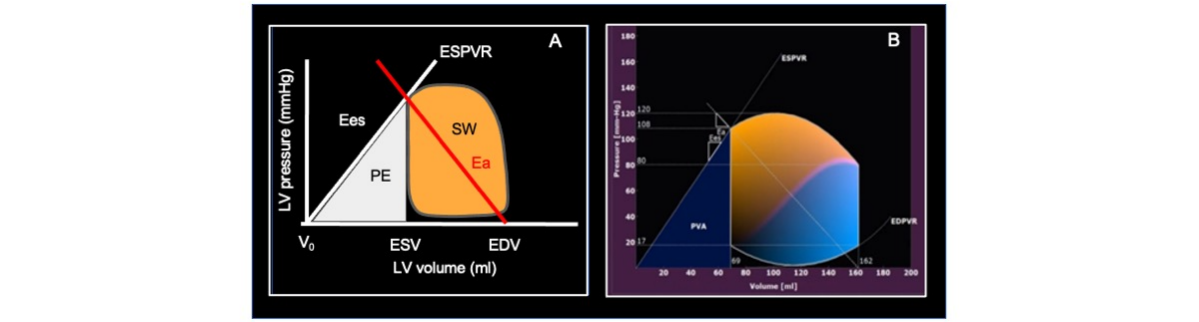
(A) Schematic representation of a pressure-volume loop. (B) Example of pressure-volume loop loop derived from echocardiographic images in one representative subject. Ea: arterial elastance; EDPVR: end-diastolic pressure-volume relation; EDV: end-diastolic volume; Ees: left ventricular systolic elastance; ESPVR: end-systolic pressure-volume relation; ESV: end-systolic volume; PE: potential energy; PVA: pressure-volume area; SW: stroke work.

LV volumes, estimated end-diastolic pressure, and brachial systolic and diastolic pressures will be used as input. The software reconstructs the pressure-volume (P-V) loop by determining the end-systolic pressure-volume relationship (ESPVR) and end-diastolic pressure-volume relationship (EDPVR) using the single-beat algorithms previously described in the literature [19]. Once the EDPVR and ESPVR are identified, the end-diastolic and end-systolic LV volumes and systolic and diastolic brachial pressures will be used to close the P-V loop. Finally, the P-V relation will be depicted for the entire cardiac cycle where each point of the curve is described as (Vt, Pt). In the P-V loop, the classic phases of the cardiac cycle are displayed: isovolumetric contraction, ejection, isovolumetric relaxation, and diastolic filling (Figure 2).

Based on this integrated P-V loop, the hemodynamic parameters to be calculated, both before and after the HD session, are listed in Textbox 3.

The pre- and posthemodialysis changes will be calculated and considered as potential prognostic indicators.

**Parameters derived from the pressure-volume loop and collected for analysis.**

- Left ventricular systolic elastance: reflecting ventricular contractility and represented by the slope of the end-systolic pressure/volume (P-V) relation.
- Arterial elastance: reflecting the effective arterial afterload and represented by the slope of the line connecting the end-diastolic volume on the volume axis to the end-systolic P-V point on the P-V loop.
- Ventricular-arterial coupling: calculated as the ratio between arterial elastance and left ventricular systolic elastance.
- Stroke work (SW): the external work performed by the myocardium to eject blood, computed as the area enclosed by the P-V loop.
- Potential energy: ventricular energy dissipated during isovolumetric contraction.
- Pressure-volume area: the total mechanical energy generated by the contraction of the left ventricle, equal to the sum of potential energy and SW.
- Work efficiency: the efficiency of the mechanical energy transfer from the ventricle to the arterial tree, expressed as the ratio between SW and pressure-volume area.

### Data Management

This research involves a medical record review, image acquisition and analysis, and data analysis. Each participant will be assigned an ID number and all hard copy data collected or used during this study will be stored in a cabinet in the locked office of the study personnel. All electronic data will be stored on password-protected computers of the study personnel in their locked offices and the locked personnel building. Only essential research personnel will have access to any of these patient files.

### Statistical Analyses

Statistical analysis will be performed with Statistical Package Stata (StataCorp) and R-studio (Posit). All continuous variables will be presented as mean (SD) or median (IQR) based on their distribution. Paired comparisons of continuous variables will be performed with a 2-tailed paired Student *t* test or Wilcoxon test, when appropriate. Paired comparisons of categorical variables will be conducted with the McNemar test. Event-free survival for each of the echocardiographic and VAC parameters will be assessed using Kaplan-Meier analysis and compared with a log-rank test, where each index will be dichotomized according to the median of its distribution. The prognostic value will be determined using the Cox proportional hazards model. Differences will be considered statistically significant when *P*<.05. Interclass correlation coefficients (ICC) will be calculated to assess the inter- and intraobserver agreement of P-V loop measurements.

### Ethical Considerations

The study was approved by the Nazarbayev University Institutional Research Ethics Committee in July 2024 (Multimedia Appendix 4), and secured funding in August 2024, after a peer-reviewed process (Multimedia Appendix 5).

One of the physicians at the dialysis center will explain the aim of the study and the echocardiographic procedure. Written informed consent will be obtained from all participants before the procedure (Multimedia Appendix 6).

Participation in the study is on a voluntary basis, thus no compensation will be offered to patients.

Important protocol modifications during the study will be submitted to the Institutional Research Ethics Committee of Nazarbayev University for approval.

## Results

### Overview

At least 323 patients are required to estimate the incidence of cardiovascular events with 95% CI and a 5% margin of error. Data collection is scheduled to occur between January 1, 2025, and June 30, 2025. As of March 18, 2025, a total of 32 participants have been recruited, who underwent echocardiogram before and after the HD session. Data analysis is planned to start in April 2025 and continue through June 2026, with certain data being collected concurrently during the analysis phase. The publication of study outcomes is tentatively projected in Winter 2027.

### Our Experience

**Figure 3:**
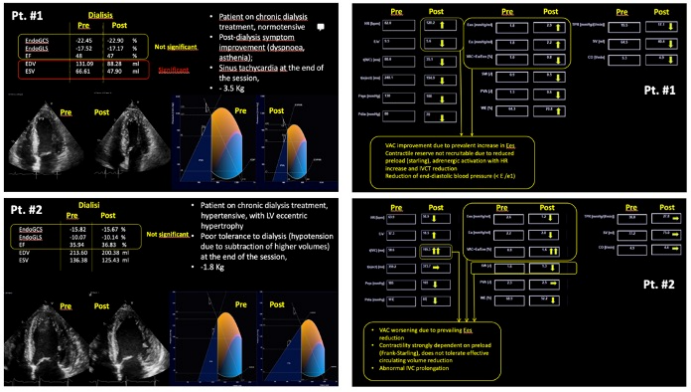
Illustrative cases of 2 patients (Pt #1 at the top, Pt #2 at the bottom) with VAC analysis before and post dialysis.

Our group has an established experience in advanced echocardiography and image postprocessing. We have applied strain and hemodynamic forces analysis in athletes and patients with hypertension [20]. Our group has already applied this method, including VAC analysis, in a preliminary group of patients with ESRD on maintenance HD. Two illustrative cases are represented in Figure 3.

Patient #1 had 3.5 liters of fluid subtraction during the HD session with VAC improvement due to an increase in Ees (from 1.8 to 2.9 mm Hg/mL). The contractile reserve was not recruitable due to reduced preload (reduced stroke volume) and the patient had sinus tachycardia at the end of the HD session due to adrenergic activation. Patient #2 had 1.8 L of fluid subtraction, with poor tolerance and symptoms at the end of the HD session. The analysis of echocardiographic images revealed VAC worsening due to Ees decrease (from 2.6 to 1.2 mm Hg/mL). In this case, contractility is strongly dependent on preload, thus the LV did not tolerate the effective circulating volume reduction.

## Discussion

### Principal Findings

Data on the value of VAC in patients on HD are scanty and controversial. Moreover, no research on this topic has been conducted in the Republic of Kazakhstan. The results of this study will provide insights into the hemodynamic changes induced by HD in patients with ESRD and will improve the understanding of the pathophysiological events induced by acute volume depletion. The prognostic value of HD-induced VAC changes will be also assessed at follow-up.

### Comparison to Previous Work

The association between myocardial and arterial function markers has been used to describe and quantify VAC in hypertension [21-23], diabetes [24], and inflammatory diseases [25]. More specifically, combined ventricular and arterial stiffening has been demonstrated in patients with heart failure with preserved ejection fraction (HFpEF) [26]. In patients with heart failure, abnormal VAC is implicated in the pathophysiology of left and right heart clinical deterioration [27]. In addition, different patterns of abnormal VAC are recognized in heart failure with reduced ejection fraction versus HFpEF. The results of these studies suggest that in hypertension and diabetes, there is a close link between arterial stiffness and myocardial deformation, which appears to be a harbinger of HFpEF if left untreated. In addition, there is evidence that treatment options may improve both myocardial dysfunction and vascular stiffness and thus improve VAC. Systemic inflammatory processes and oxidative stress may cause an increase in arterial stiffness (leading to an increase in Ea) in parallel to myocardial stiffening (causing an increase in Ees), while anti-inflammatory treatment improves arterial stiffness in parallel with myocardial stiffness contributing to the reversal of ventricular-arterial decoupling.

VAC assessment has prognostic value in several clinical scenarios. Valvulo-arterial impedance can specifically describe the hemodynamic consequences of aortic stenosis and provide prognostic information in patients with aortic stenosis even when asymptomatic [28]. There is also evidence that VAC is associated with outcomes in patients with sepsis or after myocardial infarction [29,30].

In patients with renal dysfunction, increased arterial stiffness coupled with a matched increase in ventricular stiffness has been reported [31,32]. These findings have important hemodynamic consequences as a stiff heart-arterial system generates more systolic pressure changes for a given stroke volume.

Sasso et al [33] evaluated 15 patients on HD. They concluded that HD influences both Ees and Ea/Ees ratio acutely, although this effect seems due only to the acute reduction in preload and blood pressure, without any clinically significant effect on intrinsic LV contractility and VAC. In a study on 234 HD patients, Obokata et al [34] found that Ea/Ees ratio was independently associated with adverse outcomes, providing an incremental prognostic value over the clinical score and left ventricular ejection fraction. Recently, Zuo et al [35] used an approach similar to ours (pre- and posthemodialysis assessment of VAC). They concluded that VAC is a reliable index for stratifying the risk of cardiovascular diseases in HD patients.

### Strengths and Limitations

The approach we are planning to use in this study allows a noninvasive assessment of the cardiovascular efficiency and the changes induced by volume changes during HD in patients with ESRD. The endocardial borders, evaluated by echocardiographic speckle tracking, can be used directly as input to the present model to provide a noninvasive assessment of VAC. Using standard echocardiographic images and advanced image analysis, we will be able to evaluate the heart-arterial system interaction from the VAC. We believe that, using this approach, a comprehensive analysis of the hemodynamic effects of volume changes induced by HD will be feasible. This will allow a better understanding of the pathophysiological changes induced by HD, with the potential of tailoring the optimal dialysis modalities and identifying those patients who are at higher risk of LV remodeling and future cardiovascular events.

Some challenges of this approach can be anticipated. First, echocardiographic image quality may affect proper visualization and tracking of the LV endocardial border. Second, a low frame rate can hamper the temporal resolution of the method. Finally, inter- and intraobserver reliability should be high to exclude changes due to variability.

### Future Directions

Further research should investigate whether modifying VAC through targeted interventions could improve clinical outcomes in HD patients. Expanding the study to larger, multicenter cohorts would enhance the generalizability of our findings. In addition, incorporating advanced imaging techniques to assess VAC dynamically over time may provide deeper insights into its prognostic role in patients undergoing chronic HD.

### Dissemination Plan

The final results will be presented at the main international nephrology and cardiology meetings and conferences. The development and findings of the project will be reported in relevant international journals.

### Conclusions

In conclusion, this research protocol outlines the process of noninvasive assessment of VAC before and after a HD session in patients with ESRD. The methodology we are applying does not require complex and sophisticated tools apart from the analysis software. We believe this approach will provide additional information to the relevant stakeholders and assist other researchers in developing similar studies in different clinical settings.

## Data Availability

The datasets generated or analyzed during this study are available from the corresponding author on reasonable request.

## Appendix

Multimedia Appendix 1 Explanation of the echocardiographic exam in three languages (Kazakh, Russian and English). This will help patients to understand the nature of the procedure.

Multimedia Appendix 2 Case report form used in the study.

Multimedia Appendix 3 SPIRIT (Standard Protocol Items: Recommendations for Interventional Trial) checklist.

Multimedia Appendix 4 Approval of the study from the Nazarbayev University Institutional Research Ethics Committee (IREC).

Multimedia Appendix 5 Peer review report from the Ministry of Sciences and Higher Education of the Republic of Kazakhstan.

Multimedia Appendix 6 Informed consent.

## Abbreviations

Ea: arterial elastance
EDPVR: end-diastolic pressure-volume relationship
Ees: ventricular elastance
ESPVR: end-systolic pressure-volume relationship
ESRD: end-stage renal disease
HD: hemodialysis
HFpEF: heart failure with preserved ejection fraction
ICC: Interclass correlation coefficients
LV: left ventricle
P-V: pressure-volume
SPIRIT: Standard Protocol Items: Recommendations for Interventional Trial
VAC: ventriculo-arterial coupling
