# Supplementary material for: Intradialytic Changes and Prognostic Value of Ventriculo-Arterial Coupling in Patients With End-Stage Renal Disease: Protocol for an Observational Prospective Trial
Source: JMIR Res Protoc. 2025 Jun 23;14:e71948. doi: 10.2196/71948 (PMC12235198; doi:10.2196/71948)
Supplement: Multimedia Appendix 1 [file resprot_v14i1e71948_app1.pdf]

WELCOME!

Thank you for accepting to participate in the research study on **Ventriculo-Arterial Coupling in Patients with End-Stage Renal Disease on Hemodialysis: Intra-Dialytic Changes and Prognostic Value.**

As part of this study, we will acquire some images of your heart using an echocardiographic machine.

- **What is an echocardiogram (Cardiac Ultrasound):**
  - This test uses **sound waves** (ultrasound) to create images of your heart and evaluate how well it is functioning.
  - The procedure is **painless**, and you will feel no discomfort.
  - There is **no radiation exposure**, as it uses sound waves only.
  - There are **no side effects or complications**.

- **Procedure:**
  - **A limited echocardiogram** will be performed **before and after** your hemodialysis session.
  - The echocardiogram will be **focused** on a portion of your heart (left ventricle).
  - A doctor will acquire some images of your heart using a standard machine while you lie down comfortably.
  - The images will be saved and analyzed later.
  - The test will take about **15 to 30 minutes**.

- **Echocardiogram report:**
  - Please note that **this is not a complete echocardiogram**, but only a few images of your heart will be acquired.
  - The images will be used for research purposes only.
  - Therefore, the results of the study (images and report) **will not be provided** to you as part of this study.

ҚОШ КЕЛДІҢІЗ!

Гемодиализдегі Терминалдық Бүйрек Ауруы бар науқастардағы Вентрикулярлық-Артериалды Байланыс: Гемодиализ кезіндегі Өзгерістер мен Болжау Мәні туралы зерттеу жұмысына қатысуға келісім бергеніңіз үшін рахмет.

Бұл зерттеу аясында біз сіздің жүрегіңізді эхокардиографиялық аппарат арқылы бейнелеп суреттер аламыз.

- **Эхокардиограмма (жүрек ультрадыбыстық зерттеуі) дегеніміз не:**
    - Бұл тест жүрегіңіздің суреттерін жасау үшін **дыбыстық толқындарды** (ультрадыбыс) пайдаланып, оның қалай жұмыс істейтінін бағалайды.
    - Процедура **ауыртпалықсыз**, сіз ешқандай ыңғайсыздық сезінбейсіз.
    - **Радиация әсері жоқ**, себебі тек дыбыстық толқындар қолданылады.
    - **Жанама әсерлер немесе асқынулар жоқ**.

- **Процедура:**
    - **Шектеулі эхокардиограмма** гемодиализ сессияңыздан **бұрын және кейін** жасалады.
    - Эхокардиограмма жүрегіңіздің бір бөлігіне (сол жақ қарынша) бағытталады.
    - Дәрігер сіздің жүрегіңіздің суреттерін алу үшін стандартты апарат пайдаланады, ал сіз өзіңізге жайлы күйде жатасыз.
    - Суреттер сақталып, кейін талданады.
    - Процедура шамамен **15-30 минутқа** созылады.

- **Эхокардиограмма туралы есеп:**
    - Назар аударыңыз, **бұл толық эхокардиограмма емес**, тек жүрегіңіздің бірнеше суреті алынады.
    - Суреттер тек зерттеу мақсатында қолданылады.
    - Сондықтан зерттеу нәтижелері (суреттер мен есеп) осы зерттеу аясында **сізге берілмейді**.

ДОБРО ПОЖАЛОВАТЬ!

Спасибо, что согласились участвовать в исследовательском проекте, посвященном “**Левожелудочково-Артериальное Взаимодействие у пациентов с Терминальной Стадией Почечной Недостаточности, находящихся на Гемодиализе: Внутри-Диализные изменения и Прогностическая Ценность**”.

В рамках этого исследования мы получим несколько изображений вашего сердца с помощью эхокардиографического аппарата.

- **Что такое эхокардиограмма (ультразвуковое исследование сердца):**
  - Этот тест использует **звуковые волны** (ультразвук) для создания изображений вашего сердца и оценки того, насколько хорошо оно функционирует.
  - Процедура **безболезненная**, вы не почувствуете дискомфорта.
  - **Нет облучения**, так как используются только звуковые волны.
  - **Нет побочных эффектов и осложнений.**

- **Процедура:**
  - **Ограниченная эхокардиограмма** будет выполнена **до и после** вашей сессии гемодиализа.
  - Эхокардиограмма будет **сосредоточена** на части вашего сердца (левый желудочек).
  - Врач сделает несколько изображений вашего сердца с помощью стандартного аппарата, пока вы будете лежать удобно.
  - Изображения будут сохранены и проанализированы позже.
  - Тест займет **от 15 до 30 минут**.

- **Отчет по эхокардиограмме:**
  - Обратите внимание, что **это не полная эхокардиограмма**, а только несколько изображений вашего сердца, которые будут получены.
  - Изображения будут использованы исключительно для научных целей.
  - Следовательно, результаты исследования (изображения и отчет) **не будут предоставлены** вам в рамках этого исследования.
