# Supplementary material for: Intradialytic Changes and Prognostic Value of Ventriculo-Arterial Coupling in Patients With End-Stage Renal Disease: Protocol for an Observational Prospective Trial
Source: JMIR Res Protoc. 2025 Jun 23;14:e71948. doi: 10.2196/71948 (PMC12235198; doi:10.2196/71948)
Supplement: Multimedia Appendix 2 [file resprot_v14i1e71948_app2.docx]

**SECTION 2. Patient Information**

**DEMOGRAPHIC VARIABLES**

**Date of birth (dd/mm/yyyy):** |___|___| |___|___| |___||___||___||___|

day month year

**Age:** |___|___|

**Gender: □** Male **□** Female

**VITAL SIGNS**

**• Dry weight:** |_____|_____|____|.|_____| kg

**• Height**: |_____|_____|____| cm

**• BMI** |_____|_____|____|.|_____| kg/m²

**COMORBIDITIES**

- Hypertension
- Diabetes mellitus
- Dyslipidemia
- Smoking history
- Prior myocardial infarction
- Coronary artery disease
- Revascularization
- Atrial fibrillation
- Prior HF hospitalization
- Cancer history
- Heart Failure, stage ____

**HISTORY AND MANAGEMENT OF KIDNEY DISEASE**

- **Primary diagnosis and ICD code:**

______________________________________________________________________________________________________________________________________

- **Causes of End-Stage Renal Disease (ESRD):**
- Hypertension
- Diabetes mellitus
- Glomerulonephritis
- Polycystic kidney disease
- Tubulointerstitial nephritis
- Unknown
- Other: ______________________
- **Dialysis Duration:**
  - **Start Date:** |___|___| |___|___| 2 0 |___||___|

day month year

- - **Duration:**  |___|___| years |___|___| months
- **Vascular Access:**
  - **Type:**
- Arteriovenous fistula (AVF)
- Arteriovenous graft (AVG)
- Central venous catheter (CVC)
  - **If CVC, indicate the type of the catheter (by duration):**
- Temporary catheter
- Permanent (tunneled) catheter
  - **If CVC, indicate the Location:**
- Subclavian vein
- Jugular vein
- Femoral vein
- **Hemodialysis Session (indicate the date when heart ultrasound was performed:**

|___|___| |___|___| 2 0 |___||___|

day month year

- **Hemodialysis schedule: (indicate hemodialysis schedule and circle the exact day when the heart ultrasound was performed. If Patient has different schedule, please write down the exact schedule and circle the day when the heart ultrasound was performed):**
  - If 3 sessions per week:
- Monday/Wednesday/Friday
- Tuesday/Thursday/Saturday
- ________________________________________________________________________________________________________________
- **Residual urine output:** |_____|_____|____|.|_____| mL/day
- **Actual weight before hemodialysis session:** |_____|_____|____|.|_____| kg
- **Actual weight after hemodialysis session:** |_____|_____|____|.|_____| kg
- **Date of previous hemodialysis session:** |___|___| |___|___| 2 0 |___||___|

day month year

- **Actual weight after previous hemodialysis session:** |_____|_____|____|.|_____| kg
- **Interdialytic weight gain** [actual weight before session – weight after previous HD session]: |_____|_____|____|.|_____| kg
- **Ultrafiltration volume:** |_____|_____|____|.|_____| mL
- **Blood flow rate:** |_____|_____|____|.|_____| mL/min
- **Heart rate before hemodialysis session:** |_____|_____|____| bpm
- **Heart rate during hemodialysis session:** |_____|_____|____| bpm
- **Heart rate after hemodialysis session:** |_____|_____|____| bpm
- **Blood pressure before hemodialysis session:** _________/________ mm Hg
- **Blood pressure after hemodialysis session:** _________/________ mm Hg
- **Complications during hemodialysis session (unstable hemodynamics, arrhythmia, etc.):** ____________________________________________________

___________________________________________________________________

**Laboratory data before HD session**

**Date:** |___|___| |___|___| |___||___||___||___|

day month year

| **Laboratory data** | **Values** |
| --- | --- |
| 1. **Serum creatinine** (µmol/L) |  |
| 1. **Urea** (mmol/L) |  |
| 1. **Sodium** (mEq/L) |  |
| 1. **Potassium** (mEq/L) |  |
| 1. **Albumin** (g/L) |  |
| 1. **Total calcium** (mmol/L) |  |
| 1. **Phosphate** (mmol/L) |  |
| 1. **Ferritin** (ng/L) |  |
| 1. **Hemoglobin** (g/L) |  |
| 1. **Parathyroid hormone** (ng/L) |  |
| 1. **WBC** (*10^9^/L) |  |
| 1. **TIBC** (umol/L) |  |
| 1. **Transferrin** (g/L) |  |

**List of Medications (mark and list)**

- ACEi/ARB _______________________________________________
- Beta blockers ____________________________________________
- CCB ___________________________________________________
- Statins _________________________________________________
- Antiplatelets _____________________________________________
- Diuretics ________________________________________________
- Phosphate binders ________________________________________
- Other ___________________________________________________

________________________________________________________

________________________________________________________

PHYSICIAN’S NAME: __________________________________

PHYSICIAN'S SIGNATURE: ____________

Date: |___|___| |___|___| 2 0 |___||___|

day month year

**OUTCOMES (during 18 months of follow-up)**

- **Hospitalizations:**

Number: _____

1. Date: |___|___| |___|___| 2 0 |___||___|

day month year

- **Cause of hospitalization**

__________________________________________________________________________________________________________________________________________

_____________________________________________________________________

_____________________________________________________________________

2. Date: |___|___| |___|___| 2 0 |___||___|

day month year

- **Cause of hospitalization**

__________________________________________________________________________________________________________________________________________

_____________________________________________________________________

_____________________________________________________________________

3. Date: |___|___| |___|___| 2 0 |___||___|

day month year

- **Cause of hospitalization**

__________________________________________________________________________________________________________________________________________

_____________________________________________________________________

_____________________________________________________________________

- **Nonfatal myocardial infarction**
- Yes
- No

Date: |___|___| |___|___| 2 0 |___||___|

day month year

- **Major non-cardiovascular events**

____________________________________________________________________

_____________________________________________________________________

Date: |___|___| |___|___| 2 0 |___||___|

day month year

- **Death:**

Date: |___|___| |___|___| 2 0 |___||___|

day month year

- **Cause of death:**

_____________________________________________________________________

_____________________________________________________________________

_____________________________________________________________________

*I confirm that the data obtained is accurate, complete, and consistent with the original information, and that all entries have been made either by me or by someone under my supervision.*

PHYSICIAN’S NAME: __________________________________

PHYSICIAN'S SIGNATURE: ____________

Date: |___|___| |___|___| 2 0 |___||___|

day month year

Check the following items:

1. The cover page is fully completed: □ Yes □ No
2. Informed consent has been obtained: □ Yes □ No
3. The center and patient codes are indicated on each page: □ Yes □ No
4. Patient information is fully completed: □ Yes □ No
5. The scale is fully completed, except for missing data: □ Yes □ No
6. The doctor's name, signature, and date of CRF completion are indicated: □ Yes □ No

**THANK YOU FOR PARTICIPATING IN THE STUDY!**
