# Supplementary material for: Intradialytic Changes and Prognostic Value of Ventriculo-Arterial Coupling in Patients With End-Stage Renal Disease: Protocol for an Observational Prospective Trial
Source: JMIR Res Protoc. 2025 Jun 23;14:e71948. doi: 10.2196/71948 (PMC12235198; doi:10.2196/71948)
Supplement: Multimedia Appendix 4 [file resprot_v14i1e71948_app4.pdf]

NAZARBAYEV UNIVERSITY

Autonomous organization of education
Nazarbayev University

Extract from the Decision of
Nazarbayev University
Institutional Research Ethics Committee Minutes
For the Expedited Review
№ 2 – October 31, 2024

This letter will serve as a confirmation that **Alessandro Salustri's** research project entitled **"Ventriculo-arterial coupling in patients with end-stage renal disease on hemodialysis: Intra-dialytic changes and prognostic value"** has been approved under the expedited category of review by the Nazarbayev University Institutional Research Ethics Committee (NU IREC). The conditions and duration of this approval are specified in the NU IREC Procedures.

Date of Approval: October 27, 2024
Date of Expiration: October 26, 2025
NU IREC №: 956/08102024

NU IREC Secretary ____________ Dilara Sarbassova, Head of Research Compliance and Integrity Office/ Office of Provost
