# Supplementary material for: Intradialytic Changes and Prognostic Value of Ventriculo-Arterial Coupling in Patients With End-Stage Renal Disease: Protocol for an Observational Prospective Trial
Source: JMIR Res Protoc. 2025 Jun 23;14:e71948. doi: 10.2196/71948 (PMC12235198; doi:10.2196/71948)
Supplement: Multimedia Appendix 5 [file resprot_v14i1e71948_app5.pdf]

ЗАКЛЮЧЕНИЕ ГОСУДАРСТВЕННОЙ НАУЧНО-ТЕХНИЧЕСКОЙ ЭКСПЕРТИЗЫ

ИРН AP23490021 - «Желудочково-артериальная взаимосвязь у пациентов с терминальной стадией почечной недостаточности, находящихся на гемодиализе: внутридиализные изменения и прогностическая ценность.»

Наименование критериев оценки		Средний балл (от 0 до 9)	Комментарии всех экспертов с ответами на наводящие вопросы		
1	Новизна, актуальность и перспективность проекта	8.33	The project is very novel and relevant as the applicants' aim to evaluate the effect of volume changes induced by hemodialysis on ventriculo-arterial coupling (VAC) computed from speckle-tracking echocardiography among patients with end-stage renal disease (ESRD); in particular, the study focuses on a comprehensive clinical assessment of intra-dialytic volume changes among ESRD patients undergoing hemodialysis and its prognostic significance in Kazakhstan. The main advantage of the study is the ability to utilize the clinical, and echocardiographic parameters towards a personalized approach to hemodialysis care and to potentially identify individuals at risk of complications. The hypothesis, ideas and expected results proposed in the study are interesting both nationally and internationally. Also, the scientific and/or methodological problem areas to be studied is innovative and it is an area that has not been adequately researched in the country and globally. Second, the applicants' research plan are modern, novel and interdisciplinary. The authors were able to design a study that combines clinical and imaging assessments towards better prognostication of ESRD patients receiving hemodialysis. The approaches are modern and relevant and this followed adequate literature	This project aimed at evaluating the effect of volume changes induced by hemodialysis (HD) on ventriculo-arterial coupling (VAC) computed from speckle-tracking echocardiography recruiting Three-hundred-eighty-four patients presenting with end-stage renal disease (ESRD). This is a very important objective given the clinical and social impact of myocardial and renal diseases. The relevance of the project is high particularly because data on the value of VAC in patients on hemodialysis are still controversial and no significant research data are available in the Republic of Kazakhstan. Hypotheses, ideas and expected research results are novel and methodological approaches are adequate.	Научная новизна: Данные о значении желудочково-артериальной взаимосвязи (VAC) у пациентов на гемодиализе скудны и противоречивы. В частности, исследований на эту тему в Казахстане не проводилось. Предлагаемое исследование будет первым, которое систематически оценит влияние гемодиализа на параметры VAC, включая систолическую эластичность левого желудочка (Ees) и эластичность артерий (Ea), с использованием расширенной эхокардиографии и анализа кривой соотношения давление-объем (PV loop). Новизна исследования заключается в применении современных методов анализа VAC и спекл-трекинг эхокардиографии для оценки изменений сердечной механики у пациентов с терминальной стадией почечной недостаточности (ТПН), находящихся на гемодиализе. Актуальность: Учитывая высокий риск сердечно-сосудистых заболеваний и смертности среди пациентов на гемодиализе, понимание взаимодействия сердца и сосудистой системы имеет критическое значение. Результаты исследования могут привести к улучшению клинической практики путем разработки новых протоколов мониторинга и лечения, направленных на снижение риска

ЭКСПЕРТНОЕ ЗАКЛЮЧЕНИЕ
АО "НАЦИОНАЛЬНЫЙ ЦЕНТР ГОСУДАРСТВЕННОЙ
НАУЧНО-ТЕХНИЧЕСКОЙ ЭКСПЕРТИЗЫ"

			review.		сердечно-сосудистых событий у пациентов с ТПН. Актуальность проекта подтверждается потребностью в новых данных для разработки эффективных методов оценки и прогнозирования неблагоприятных исходов у пациентов на гемодиализе. Эти аспекты подчеркивают важность исследования как для научного сообщества, так и для улучшения клинической практики в области нефрологии и кардиологии.
			The project intends to address an important clinical challenge i.e. intravascular volume changes and its effects among patients with end-stage renal disease (ESRD) undergoing hemodialysis. Globally, it is estimated that 4.9–9.7 million people have ESRD were estimated to require renal replacement therapy. Hemodialysis causes rapid volume shifts and circulatory changes. The risk of cardiovascular mortality in dialysis patients is approximately 9 times higher than that of the general population, and young dialysis patients were characterized by extraordinarily high risk. Dialysis-induced hemodynamic instability is one of the most common complications, and those patients with unstable hemodynamics during hemodialysis were associated with worse outcomes. The project is of immense scientific and technical level and will extend the knowledge on dialysis care in Kazakhstan. The project will not only clarify volume changes induced by hemodialysis on ventriculo-arterial coupling (VAC) computed from speckle-tracking echocardiography among patients with	Approaches and methods used in this project are modern and relevant, the applicants possess strong expertise in echocardiographic speckle tracking images, advanced echocardiography and image post-processing, and relative software able to reconstruct the pressure/volume (PV) loop by determining the end-systolic and end-diastolic pressure-volume relationship using the single-beat algorithms. Moreover, literature referred to by the authors of the project is relevant.	Важность проекта: Исследование желудочково-артериальной взаимосвязи (VAC) у пациентов с терминальной стадией почечной недостаточности (ТПН), находящихся на гемодиализе, является критически важным для понимания и улучшения их сердечно-сосудистого здоровья. Проект предоставляет уникальные возможности для внедрения новых диагностических методов и улучшения клинических протоколов на основе полученных данных. Учитывая высокий уровень смертности и частоту сердечно-сосудистых заболеваний у пациентов на гемодиализе, результаты исследования могут значительно повысить качество жизни и выживаемость этой группы пациентов. Актуальность научно-технического уровня: Проект использует передовые методы, такие как спекл-трекинг эхокардиография и анализ кривой соотношения давление-объем (PV loop), которые предоставляют более точные и надежные данные о состоянии сердечно-сосудистой системы. Применение этих методов

ЭКСПЕРТНОЕ ЗАКЛЮЧЕНИЕ
АО "НАЦИОНАЛЬНЫЙ ЦЕНТР ГОСУДАРСТВЕННОЙ НАУЧНО-ТЕХНИЧЕСКОЙ ЭКСПЕРТИЗЫ"

			ESRD in the country, but will also explore the relationship between intra-dialytic volume changes and prognosis of the hemodialysis. The resulting data will be a valuable source for identifying unique diagnostic and prognostic opportunities, which will significantly improve the quality care given to ESRD patients and identify specific factors associated with cardiovascular mortality in dialysis patients. Therefore, the hypotheses, ideas and expected results of the research are promising and would be of interest worldwide. The project is capable of being a major breakthrough for science in the country. The findings of this project would be considered for publication in highly rated journals. In addition, the quality and quantity of publications is adequate. The project has proposed adequate number of publications, and the publications planned as well as the target journals as the project outcome meet the requirements of the call documentation. In addition, the number of papers planned for publication in the proposal is reasonable and achievable.		позволяет детально оценить изменения сердечной механики и выявить потенциальные риски, связанные с гемодиализом. Научно-технический уровень проекта соответствует международным стандартам и включает в себя новейшие технологии в области кардиологии и нефрологии. Степень разработанности проекта: Проект основывается на предыдущих исследованиях и опыте исследовательской группы в области расширенной эхокардиографии и постобработки изображений. Исследовательская группа уже применяла данный метод на предварительной группе пациентов с ТПН, что подтверждает его жизнеспособность и применимость. В проекте участвуют высококвалифицированные специалисты с международным опытом, что обеспечивает высокий уровень выполнения и интерпретации результатов. Развитие науки и техники: Проект способствует развитию новых диагностических подходов и методов оценки сердечно-сосудистой системы у пациентов на гемодиализе. Результаты исследования могут стать основой для дальнейших исследований и разработок в области кардиологии и нефрологии. Научно-технические достижения проекта могут быть использованы для разработки новых медицинских устройств и программного обеспечения, что повысит точность и эффективность диагностики и лечения сердечно-сосудистых заболеваний.

ЭКСПЕРТНОЕ ЗАКЛЮЧЕНИЕ
АО "НАЦИОНАЛЬНЫЙ ЦЕНТР ГОСУДАРСТВЕННОЙ НАУЧНО-ТЕХНИЧЕСКОЙ ЭКСПЕРТИЗЫ"

					Заключение: Проект обладает значительной важностью и актуальностью, внедряя передовые научно-технические методы и способствуя развитию науки и техники. Высокая степень разработанности и участие квалифицированных специалистов обеспечивают его успешное выполнение и потенциально значимый вклад в медицинскую практику и науку.
2	Качество и реализуемость исследовательского плана	7.66	The research is well designed. The authors proposed a clearly defined, well-substantiated, quality research work plan which involves key steps like reviewing the current literature, sampling and acquisition of echocardiographic images, off-line analysis of echocardiographic images, non-invasive pressure-volume loop analysis, outcome assessment and preparation and submission of the report and recommendations. Given that this is an emerging area of scientific interest, the applicants' adequately substantiated and justified the research problem in their research plan. Moreover, the applicants' had clear and quality research plan in formulating the aims, objectives and hypothesis of the research. The applicants' planned to investigate clinical assessment of intra-dialytic volume changes among ESRD patients undergoing hemodialysis and its prognostic significance in Kazakhstan. The project has very clear aims, objectives and hypotheses of the research plan. Moreover, the hypothesis proposed is good and realistic.	The quality of the research plan is very good and clinical problems are well highlighted. Patients will be followed-up for 18 months and the project is characterized by a very good likelihood to achieve its goals since it shows to be well articulated concerning the time frame, the coherence among the proposed activities cost and the justification of the proposed budget. In fact, the relative amounts for the cost of activities concerning: staff, contract with young researchers, equipment, personnel travel, subcontractors and overheads, are balanced as well.	Исследовательский план проекта характеризуется высокой степенью структурированности и научной обоснованности. Проект включает комплексный подход к изучению желудочково-артериальной взаимосвязи (VAC) у пациентов с терминальной стадией почечной недостаточности (ТПН), находящихся на гемодиализе, с использованием передовых методов, таких как спекл-трекинг эхокардиография и анализ кривой соотношения давление-объем (PV loop). Исследовательский план предусматривает: Тщательный обзор литературы и существующих данных. Систематический сбор эхокардиографических данных до и после сеансов гемодиализа. Оффлайн анализ изображений с использованием специализированного программного обеспечения. Долгосрочное наблюдение за пациентами для оценки клинических исходов. Статистический анализ данных с применением моделей пропорциональных рисков Кокса. План тщательно продуман, включает в себя четкие этапы и

ЭКСПЕРТНОЕ ЗАКЛЮЧЕНИЕ
АО «НАЦИОНАЛЬНЫЙ ЦЕНТР ГОСУДАРСТВЕННОЙ НАУЧНО-ТЕХНИЧЕСКОЙ ЭКСПЕРТИЗЫ»

					методы, что обеспечивает высокое качество и надежность получаемых данных.
			Given the outlined project design, the research methodology adopted is fully justified in order to meet the project goals. Thus, the methodology proposed to be used is appropriate and justified without limitations. The applicants proposed a prospective and descriptive study design involving eligible patients with ESRD. The applicants plan to recruit 384 eligible ESRD patients. Each patient, data on their demographic characteristics, full medical history, and dialysis-related parameters and echocardiographic parameters during the first and second session of their weekly hemodialysis session will be recorded on purpose-built data collecting sheets assessment. The applicants' demonstrate consistency between research questions and data collection methods. Also, it is clear that all the proposed steps suggest that the data collection is been planned correctly to enable statistical processing. Although, the applicants did not identify the member of the team responsible for statistical analysis of the data, they did highlight the statistical tool and approaches they would use like: two-tailed paired Student's t test or Wilcoxon test, & survival analysis. Minimal risks are involved in all procedures. These risks are not beyond what is expected in the provision of standard clinical services. The applicants indicated where the study will receive ethical approval from a named university, and they have taken adequate steps to prevent and address any ethical issues related to the prevention of plagiarism,	The applicant demonstrates consistency between research questions and data collection methods and the experiments were very well planned for subsequent statistical processing of the data. However, there is no clear indication about issues related to the prevention of plagiarism, falsification, and fabrication of data, false co-authorship, and the assignment of results. Ethical consideration related to human study are well illustrated, all trial procedures, protocols and data collections and storage well presented. The methodology concerning data collection and results is adequately indicated.	Методология исследований в данном проекте отличается высокой степенью точности, инновационности и научной обоснованности, что обеспечивает надежность и валидность получаемых данных. Ключевые аспекты методологии включают: Спекл-трекинг эхокардиография: Использование передового метода спекл-трекинг эхокардиографии позволяет детально оценить параметры желудочково-артериальной взаимосвязи (VAC) и изменения в сердечной механике у пациентов с терминальной стадией почечной недостаточности (ТПН), находящихся на гемодиализе. Анализ эхокардиографических изображений проводится оффлайн с использованием специализированного программного обеспечения QStrain Medis BV, что обеспечивает высокую точность и воспроизводимость результатов. Анализ кривой соотношения давление-объем (PV loop): Метод включает восстановление кривой PV для всего сердечного цикла, что позволяет оценить ключевые гемодинамические параметры, такие как систолическая эластичность левого желудочка (Ees) и эластичность артерий (Ea). Интегрированный анализ PV-кривой обеспечивает всестороннее понимание изменений сердечно-сосудистой функции в ответ на гемодиализ. Долгосрочное наблюдение: Пациенты будут

ЭКСПЕРТНОЕ ЗАКЛЮЧЕНИЕ
АО "НАЦИОНАЛЬНЫЙ ЦЕНТР ГОСУДАРСТВЕННОЙ
НАУЧНО-ТЕХНИЧЕСКОЙ ЭКСПЕРТИЗЫ"

			falsification, and fabrication of data, false co-authorship, and the assignment of results.		находиться под наблюдением в течение 18 месяцев для оценки первичных и вторичных конечных точек, таких как совокупность всех причин смерти, нефатальный инфаркт миокарда и госпитализации по поводу сердечной недостаточности. Выживаемость без осложнений будет оцениваться с помощью анализа Каплана-Мейера и моделей пропорциональных рисков Кокса, что позволяет определить прогностическую ценность VAC-параметров. Статистический анализ: Использование статистических пакетов Stata и R-studio для анализа данных обеспечивает высокую точность и надежность статистических выводов. Применение парного t-теста Стьюдента, теста Уилкоксона и теста Мак-Немара для парных сравнений непрерывных и категориальных переменных соответственно. Методология проекта тщательно продумана и включает использование современных технологий и методов анализа, что обеспечивает высокое качество и надежность получаемых результатов.
			The proposed project is likely to achieve the expected results and the research results will most likely be accepted for publication in high quality journals indicated in the application. This is because the research involves a rare and innovative area of interest which utilize clinical, and echocardiographic assessment to develop a personalized approach to hemodialysis for patients with ESRD and to identify intra-	Overall, the project is characterized by a very good likelihood to achieve its goals since is well articulated, well planned concerning the time frame, the coherence among the proposed activities cost and the justification of the proposed budget. The project is competitive.	Проект обладает высокой вероятностью достижения поставленных целей благодаря тщательно разработанной методологии, квалифицированной команде исследователей и реалистичным планам по выполнению задач. Методологическая обоснованность: Использование спекл-трекинг эхокардиографии и анализа кривой соотношения давление-объем (PV

ЭКСПЕРТНОЕ ЗАКЛЮЧЕНИЕ
АО "НАЦИОНАЛЬНЫЙ ЦЕНТР ГОСУДАРСТВЕННОЙ НАУЧНО-ТЕХНИЧЕСКОЙ ЭКСПЕРТИЗЫ"

			dialytic volume changes and its potential role as prognostic factors. These innovative strategies have great implications in the prognostication of ESRD patients following hemodialysis in the country. The project has high likelihood of achieving the expected results given that this is mainly one of the early attempt to assess specific cardiovascular volume changes and hemodynamics during hemodialysis for ESRD patients in Kazakhstan. Therefore, it is very likely that the research results will be accepted for publication in high quality journals indicated in the application. There are no obvious risks beyond those involved in completing a research of this quality such as delays or inability to procure necessary scientific equipment, reagents or consumables. Besides, the applicants have taken appropriate steps to mitigate any risks given their experience and background in the field. Moreover, the proposed project and the research plan is competitive compared to alternatives given that this is an under investigated area of research both globally and in Kazakhstan.		loop) обеспечивает точность и воспроизводимость измерений, что повышает достоверность результатов. Проект включает систематический сбор данных до и после сеансов гемодиализа, что позволяет получать детальные и последовательные результаты. Квалифицированная команда: Исследовательская группа включает экспертов с международным опытом в области кардиологии, нефрологии и медицинской визуализации, что гарантирует высокий уровень выполнения исследований. Руководитель проекта, профессор Алессандро Салустри, имеет обширный опыт в области неинвазивной кардиологии и публикаций в международных рецензируемых журналах, что способствует успешной реализации проекта. Реалистичный план выполнения: План проекта включает детальный график выполнения задач, начиная с обзора литературы и обучения исследовательской группы до сбора данных и анализа результатов. Этапы проекта расписаны по месяцам, что позволяет четко следить за прогрессом и своевременно корректировать действия при необходимости. Долгосрочное наблюдение и статистический анализ: Пациенты будут находиться под наблюдением в течение 18 месяцев, что позволяет собрать достаточное количество данных для надежных выводов. Применение моделей пропорциональных рисков Кокса и анализа Каплана-Мейера для оценки прогностической ценности

ЭКСПЕРТНОЕ ЗАКЛЮЧЕНИЕ
АО "НАЦИОНАЛЬНЫЙ ЦЕНТР ГОСУДАРСТВЕННОЙ
НАУЧНО-ТЕХНИЧЕСКОЙ ЭКСПЕРТИЗЫ"

					параметров VAC обеспечивает статистическую обоснованность результатов. Поддержка и финансирование: Проект обеспечен необходимым финансированием и ресурсами для проведения всех этапов исследований, включая приобретение оборудования и программного обеспечения. Эти факторы совместно обеспечивают высокую вероятность достижения всех поставленных целей и получение значимых научных результатов.
3	Ожидаемые результаты и их значимость	7.33	The project is very effective and efficient because the applicants has itemized the aims and objectives, well-developed hypothesis, and clear rationale, adequate review of the literature as well as appropriate methodological approaches proposed, Therefore, the expected project result is justified against the requested project funds. Given the proposed financial breakdown on how the funds will be expended, it is clear how effectively the funds will be expended to achieve the expected results of the study. According to the breakdown given, scientific and organizational support, and purchase of materials and equipment and /(or) software for the implementation of research accounted for just 37.9% of the total requested budget for the project. Also, remuneration for labor and business trips accounted for another 62.1% of the total requested funding; which is not efficient. In order to improve the efficiency and effectiveness of the research, there is a need to for the applicants' to substantially lower their remuneration for labor. There is a need for the applicants to consider	The effectiveness and efficiency of the project are coherent, the expected project results are highly significant and justify the requested funding. Overall, the results may significantly contribute to further clarify the acute effect of hemodialysis on left ventricular mechanics	Проект обладает высокой результативностью и эффективностью благодаря своей четкой научной цели, продуманной методологии, профессиональной команде исследователей и поддержке необходимыми ресурсами. Научная цель и значимость: Проект направлен на изучение желудочково-артериальной взаимосвязи (VAC) у пациентов с терминальной стадией почечной недостаточности (ТПН) на гемодиализе, что является важной и малоизученной областью. Ожидаемые результаты будут способствовать лучшему пониманию сердечно-сосудистой динамики при гемодиализе и помогут разработать новые протоколы лечения и мониторинга. Методология: Применение передовых методов, таких как спекл-трекинг эхокардиография и анализ кривой соотношения давление-объем (PV loop), обеспечивает точность и надежность данных. Систематический подход к сбору и анализу данных до и после сеансов

АО "НАЦИОНАЛЬНЫЙ ЦЕНТР ГОСУДАРСТВЕННОЙ НАУЧНО-ТЕХНИЧЕСКОЙ ЭКСПЕРТИЗЫ"

			increasing their sample size to 500 patients. This will increase the cost of materials to over 50% of the budget and the new samples size will ensure higher quality statistical analysis and will attract publications in a higher impact journals.		гемодиализа позволяет выявить важные изменения в сердечно-сосудистой системе пациентов. Профессиональная команда: Исследовательская группа состоит из высококвалифицированных специалистов с международным опытом, что гарантирует высокий уровень выполнения исследований. Руководитель проекта, профессор Алессандро Салустри, имеет обширный опыт и публикации в области кардиологии, что повышает шансы на успешное выполнение проекта. Ресурсы и поддержка: Проект обеспечен необходимым финансированием, что позволяет приобрести современное оборудование и программное обеспечение для проведения исследований. Планируются научные командировки и участие в международных конференциях, что способствует обмену знаниями и повышению квалификации исследовательской группы. Ожидаемые результаты: Проект предполагает публикацию не менее трех статей в международных рецензируемых журналах, что подтвердит его научную значимость. Результаты проекта могут быть внедрены в клиническую практику, улучшая качество лечения пациентов с ТПН на гемодиализе и снижая риск сердечно-сосудистых осложнений. Эти аспекты обеспечивают высокую результативность и эффективность проекта, способствуя значительному вкладу в науку и медицинскую практику.
			Specifically, the findings/outputs of the project will be applicable to the	The expected results of the present study (that are well indicated in the	Ожидаемые результаты проекта обладают значительной научной и

ЭКСПЕРТНОЕ ЗАКЛЮЧЕНИЕ
АО "НАЦИОНАЛЬНЫЙ ЦЕНТР ГОСУДАРСТВЕННОЙ НАУЧНО-ТЕХНИЧЕСКОЙ ЭКСПЕРТИЗЫ"

			medical and health sciences field in particular in stimulating innovation in the field of nephrology and renal medicine. The results of the study will serve as an impetus for the successful identification of whether intra-dialytic volume changes are independent predictors for poor outcomes among ESRD patients receiving hemodialysis; the identified predictors could be integrated into clinical algorithms for managing ESRD patients with hemodialysis and could be used in setting up individual treatment plans. The identified factors could be used for prognostication following hemodialysis of ESRD patients. Furthermore, in comparison with existing alternatives, the findings/outputs of the project will be competitive with other important studies in the renal sciences. There are no obvious economic benefits of the project beyond an understanding of different hemodialysis regimen on cardiac hemodynamic; and the possibility of an early detection of high-risk patients. Also, there are no practical use of the research with respect to commercialization. However, the expected research results will have many practical uses in particular with respect to education about hemodynamic changes during dialysis; as well as the development of clinical algorithms and recommendations. The papers from this project published as a result of the project will be regularly used and will be highly cited. However, inadequate number of young researchers were included in the project to enable their training. Moreover, the applicants have clearly and thoroughly	text) show high potential in terms of application in diverse areas of medical research (i.e., cardiologists, nephrologists, surgeons) and activities, and can contribute in advancing in the public health. Expected results are competitive in comparison with existing alternatives, ready for the practical-clinical application. The project may significantly contribute to further improve the evaluation of the interaction of cardiac contractility with the arterial system and provide a more comprehensive understanding of the cardiovascular function and cardiac energetics. Contracts with young researchers are well balanced and their involvement in the project is well detailed.	практической значимостью. Изучение желудочково-артериальной взаимосвязи (ЖАВ) у пациентов с терминальной стадией почечной недостаточности, находящихся на гемодиализе (ГД), позволит получить новые данные о влиянии изменений объема жидкости на сердечно-сосудистую систему. Основные результаты исследования включают оценку параметров ЖАВ до и после сеанса ГД, а также их значение для прогнозирования неблагоприятных исходов. Научная значимость Результаты исследования будут способствовать углублению понимания механики сердца и артериальной системы у пациентов с хронической почечной недостаточностью. Полученные данные позволят оценить изменения в эластичности левого желудочка (Ees) и артериальной системы (Ea), а также их взаимосвязь (VAC). Это, в свою очередь, позволит выявить патофизиологические механизмы, лежащие в основе сердечно-сосудистых осложнений у данной категории пациентов. Включение методики спекл-трекинг эхокардиографии и неинвазивного анализа кривой соотношения давление-объем (PV loop) обеспечит более точную оценку сердечно-сосудистого статуса пациентов. Практическая значимость Практическая значимость проекта заключается в возможности применения полученных данных для оптимизации схем гемодиализа и индивидуализации лечения

ЭКСПЕРТНОЕ ЗАКЛЮЧЕНИЕ
АО "НАЦИОНАЛЬНЫЙ ЦЕНТР ГОСУДАРСТВЕННОЙ НАУЧНО-ТЕХНИЧЕСКОЙ ЭКСПЕРТИЗЫ"

			demonstrated the significance of the expected results which are reliable and reasonable.		пациентов с терминальной стадией почечной недостаточности. Оценка параметров ЖАВ до и после сеанса ГД позволит выявить пациентов с высоким риском неблагоприятных сердечно-сосудистых исходов и адаптировать терапию с учетом индивидуальных особенностей. Это поможет снизить частоту госпитализаций и улучшить качество жизни пациентов. Влияние на клиническую практику Прогностическая ценность параметров ЖАВ, выявленная в ходе исследования, может быть использована для разработки новых клинических рекомендаций и протоколов ведения пациентов на гемодиализе. Это, в свою очередь, будет способствовать снижению смертности и заболеваемости среди данной группы пациентов. Внедрение методик неинвазивного мониторинга сердечно-сосудистой системы позволит улучшить контроль над состоянием пациентов и повысить эффективность проводимой терапии. Таким образом, результаты исследования не только обогатят научное сообщество новыми знаниями о взаимодействии сердца и артериальной системы при изменениях объема, вызванных гемодиализом, но и окажут значительное влияние на клиническую практику, улучшив прогноз и качество жизни пациентов с терминальной стадией почечной недостаточности.
4	Компетентность и научный задел исследовательской группы	8.33	The principal investigator/project manager who is a specialist in cardiology with long experience in advanced cardiac imaging regularly	Primary Investigator possesses strong expertise in clinical cardiology and cardiac imaging, he is board certified in internal medicine and cardiology	Профессор Алессандро Салустри, руководитель проекта, обладает высоким уровнем профессиональной квалификации и

ЭКСПЕРТНОЕ ЗАКЛЮЧЕНИЕ
АО "НАЦИОНАЛЬНЫЙ ЦЕНТР ГОСУДАРСТВЕННОЙ НАУЧНО-ТЕХНИЧЕСКОЙ ЭКСПЕРТИЗЫ"

			publishes high quality papers in renowned international journals including as the principal author (corresponding author or first author). Furthermore, the principal investigator/supervisor has published some of his papers in very highly ranked journals in which the supervisor publishes the results of his/her research. The supervisor have a long standing experience in successfully leading research projects that have published articles in peer-reviewed scientific journals and all the authors appear to have some background in the research area. The principal investigator has solid experience and scientific background in cardiology and cardiac imaging using echocardiography and has successfully led research projects that have published articles in peer-reviewed scientific journals. Also, the principal investigator has the scientific background in the form of articles and patents in this area of research.<br><br>ЭКСПЕРТНОЕ ЗАКЛЮЧЕНИЕ АО "НАЦИОНАЛЬНЫЙ ЦЕНТР ГОСУДАРСТВЕННОЙ НАУЧНО-ТЕХНИЧЕСКОЙ ЭКСПЕРТИЗЫ"	with long experience in advanced cardiac imaging, and regularly publish articles in peer-reviewed scientific journals in the research field, including as the principal author. Some of the papers have been published in very good and highly ranked Journals. The supervisor has an excellent scientific background and experience in successfully leading research projects that have published articles in peer-reviewed scientific journals.	значительным научным заделом в области кардиологии и эхокардиографии. Его академическая и исследовательская карьера включает работу в ведущих медицинских учреждениях и участие в крупных международных научных проектах. Образование и карьера Профессор Салустри является заведующим кафедрой клинических наук в Школе медицины Назарбаев Университета. Ранее он занимал должности директора отделения неинвазивной кардиологии в Медицинской корпорации Хамад (Доха, Катар), консультанта по кардиологии в Кливлендской клинике (Абу-Даби, ОАЭ) и заведующего кардиологическим отделением в Policlinico Luigi Di Liegro (Рим, Италия). Научные достижения Профессор Салустри является автором более 100 рецензируемых статей и 9 глав в книгах, а также соредактором двух книг. Его исследования охватывают широкий спектр тем, включая визуализацию сердца, расширенную эхокардиографию, новые технологии в области визуализации сердца и гипертонию. Его публикации имеют высокий импакт-фактор и цитируются в престижных научных журналах, таких как Journal of the American College of Cardiology и European Heart Journal. Проекты и гранты Руководитель проекта имеет опыт успешного привлечения грантового финансирования и реализации научных проектов. Например, грантовое финансирование МНВО РК ИРН AP14869730 для

					исследования влияния интенсивных тренировок на сердечную механику у спортсменов. Этот проект, как и текущий, направлен на использование передовых методов неинвазивной визуализации для изучения сердечно-сосудистой функции. Международное признание Профессор Салустри активно участвует в международных научных конференциях и имеет прочные связи с ведущими исследовательскими центрами и учеными по всему миру, что подтверждает его высокий научный статус и признание в профессиональном сообществе. Таким образом, высокий уровень научной квалификации, значительный опыт в области кардиологии и успешная реализация крупных научных проектов подтверждают компетентность профессора Алессандро Салустри в качестве руководителя данного проекта.
			The research team is of very high quality and they are all highly published in the field and have history of working together. Overall, the qualifications and experience of members of the research team are relevant to their roles and positions in the project. In addition, they have sufficient qualification to work on all equipment they highlighted. The project participants have sufficient qualifications to work on the materials planned to be purchased. The role of each member of the research group in the study is well substantiated and detailed in the work plan with specific activities. Indeed, the contribution of	The involved investigators (particularly the advisor who possess very strong expertise in fluid mechanics, cardiac mechanics, and clinical cardiology, inventor of several patents and actively involved in medical imaging industry) are well established in the field of this project with a relevant experience in published articles in peer-reviewed scientific journals and their background is adequate to carried out the research.	Исследовательская группа, работающая под руководством профессора Алессандро Салустри, состоит из высококвалифицированных специалистов с обширным опытом в области кардиологии, эхокардиографии и медицинской визуализации. Состав группы и квалификация Профессор Джанни Педрищетти - ведущий специалист в области биоинженерии и механики биожидкостей, профессор Университета Триеста (Италия). Автор более 150 научных работ и 10 патентов. Его исследования в области динамики сердечной

ЭКСПЕРТНОЕ ЗАКЛЮЧЕНИЕ
АО "НАЦИОНАЛЬНЫЙ ЦЕНТР ГОСУДАРСТВЕННОЙ НАУЧНО-ТЕХНИЧЕСКОЙ ЭКСПЕРТИЗЫ"

			every member of the research team as stated in the work plan is needed to complete the research following the stated goal, objectives, expected results and proposed research plan. Also, the qualifications and experience of the research team are relevant to their roles and positions in the project There is no evidence that foreign scientists / experts will participate in the project or will play any role in strategic project management, assistance in project preparation, and scientific data processing. Overall, the role of a foreign expert in this project is not justified in terms of their role in achieving the goals, objectives and expected results of the project.		жидкости и взаимодействия кровотока с тканями сердца получили широкое признание. Махаббат Бекбосынова - председатель правления АО "Национальный научный кардиохирургический центр", доктор медицинских наук. Имеет более 19 лет опыта на руководящих должностях в медицинских и научных организациях. Динара Галиева - инструктор Школы медицины Назарбаев Университета, имеет опыт участия в крупномасштабных научных проектах и высокую квалификацию в области кардиологии. Динара Джумадилова - ассистент профессора Школы медицины Назарбаев Университета, специалист по визуализации сердца и рентгенологии. Анара Аббай - кардиолог Школы медицины Назарбаев Университета, участвующая в крупномасштабных научных проектах. Научные достижения и публикации Члены исследовательской группы являются авторами множества публикаций в высокорейтинговых научных журналах, что подтверждает их высокий научный потенциал и компетентность. Их работы охватывают различные аспекты кардиологии, включая эхокардиографию, анализ гемодинамических параметров и сердечно-сосудистые заболевания. Международное сотрудничество Группа имеет прочные связи с международными научными учреждениями, такими как Университет Триеста и Калифорнийский университет в

ЭКСПЕРТНОЕ ЗАКЛЮЧЕНИЕ
АО "НАЦИОНАЛЬНЫЙ ЦЕНТР ГОСУДАРСТВЕННОЙ НАУЧНО-ТЕХНИЧЕСКОЙ ЭКСПЕРТИЗЫ"

			ЭКСПЕРТНОЕ ЗАКЛЮЧЕНИЕ АО "НАЦИОНАЛЬНЫЙ ЦЕНТР ГОСУДАРСТВЕННОЙ НАУЧНО-ТЕХНИЧЕСКОЙ ЭКСПЕРТИЗЫ"		Ирвайне. Это сотрудничество позволяет обмениваться передовыми методиками и получать доступ к новейшим научным данным и технологиям. Вовлеченность молодых ученых В проект активно вовлекаются молодые ученые, аспиранты и студенты-медики, что способствует развитию их научного потенциала и профессиональных навыков. Таким образом, исследовательская группа обладает высококвалифицированными специалистами с международным признанием, обширным опытом и значительным научным потенциалом, что обеспечивает высокое качество и успешное выполнение проекта.
			The detailed proposal and research work plan indicate the availability of resources and access to infrastructure for the successful execution of the project. Also, the infrastructure available to the applicants meet the needs of the research plan. Overall, the research equipment and other materials available to the applicants will allow the application of the proposed approaches and research methods. The role of each member of the research group in the study is well substantiated and detailed in the work plan with specific activities. Indeed, the contribution of every member of the research team as stated in the work plan is needed to complete the research following the stated goal, objectives, expected results and proposed research plan. Also, the qualifications and experience of the research team are relevant to their roles and positions in the project and	The infrastructure needed for the projects, together with the research equipment and other tools available to the applicant are well described and adequate for the objectives and scope of the project.	Исследовательская группа располагает необходимыми ресурсами и доступом к современной инфраструктуре, что обеспечивает высокое качество выполнения проекта. Оборудование и технологии Проект будет проводиться на базе Школы медицины Назарбаев Университета (ШМНУ), Национального научного кардиохирургического центра (ННКЦ) и Центра эфферентной терапии и программного гемодиализа ТОО "B.B.NURA". Эти учреждения оснащены передовым медицинским оборудованием и программным обеспечением для проведения комплексных исследований в области кардиологии и гемодиализа. Эхокардиографические системы: В ШМНУ и ННКЦ имеются коммерчески доступные эхокардиографические системы для

			complies with the research plan. The materials to be procured by the applicants in the project comply with the research plan. The project participants are all sufficiently qualified to operate the equipment and will be able to effectively use the equipment purchased, including after project completion. Therefore, the members of the research team can carry out the work themselves. Because, the equipment to be purchased had the potential to be used for future research and clinical practice, it is justified. There is no justification for using co-executives in the project and there are no co-executive included by the applicants.		проведения высокоточных ультразвуковых исследований сердца. Программное обеспечение: Используется специализированное программное обеспечение для спекл-трекинг эхокардиографии (QStrain Medis BV), что позволяет проводить детальный анализ кривых соотношения давление-объем (PV loop) и других гемодинамических параметров. Доступ к пациентам Набор пациентов будет осуществляться на базе Центра эфферентной терапии и программного гемодиализа ТОО "B.B.NURA". Центр функционирует круглосуточно и предоставляет экстренную помощь пациентам, нуждающимся в заместительной почечной терапии, что обеспечивает стабильный поток пациентов для исследования. Человеческие ресурсы В исследовательскую группу входят опытные кардиологи, рентгенологи и специалисты по визуализации сердца. Также предусмотрено участие научных сотрудников со степенью магистра, которые будут работать на полный рабочий день для содействия в отборе участников, получении и анализе изображений, сборе данных и координации логистики проекта. Международные связи Проект включает сотрудничество с международными экспертами, такими как профессор Джанни Педриццетти из Университета Триеста (Италия), что обеспечивает доступ к передовым знаниям и методологиям. Участие в международных конференциях и командировки для обучения и

ЭКСПЕРТНОЕ ЗАКЛЮЧЕНИЕ
АО "НАЦИОНАЛЬНЫЙ ЦЕНТР ГОСУДАРСТВЕННОЙ НАУЧНО-ТЕХНИЧЕСКОЙ ЭКСПЕРТИЗЫ"

					обмена опытом также предусмотрены. Финансовое обеспечение Проект имеет подробный план финансирования, включающий оплату труда научных сотрудников, расходы на командировки, приобретение материалов и оборудования, что подтверждает устойчивую финансовую поддержку. Таким образом, исследовательская группа располагает всем необходимым оборудованием, доступом к пациентам, высококвалифицированными специалистами и финансовой поддержкой для успешного выполнения проекта.
5	Междисциплинарность проекта	1.66	The project is interdisciplinary, but the approach presented in the application is not sufficiently substantiated. Therefore, the project is interdisciplinary in terms of ensuring cooperation between narrow scientific fields because it allows cooperative work between cardiologists, nephrologists, laboratory personnel as well as cardiac imaging technicians. The interdisciplinary approach is fully justified in the application and is necessary to achieve the goal of the project.	The project is interdisciplinary in terms of ensuring cooperation between broad scientific fields, an interdisciplinary approach is fully justified in the application and is necessary to achieve the goal of the project.	Проект объединяет кардиологию, нефрологию, биоинженерию и медицинскую визуализацию. Исследовательская группа включает кардиологов, специалистов по эхокардиографии и биоинженеров, что позволяет проводить комплексный анализ сердечно-сосудистых изменений у пациентов с почечной недостаточностью. Использование спекл-трекинг эхокардиографии и анализа кривых соотношения давление-объем (PV loop) требует интеграции знаний в области механики биожидкостей и клинической медицины. Сотрудничество с международными экспертами и участие в международных конференциях обеспечивают обмен передовыми методологиями и результатами, способствуя развитию междисциплинарного подхода к исследованию.
Итоговый балл (сумма баллов по		33.33			

ЭКСПЕРТНОЕ ЗАКЛЮЧЕНИЕ
АО "НАЦИОНАЛЬНЫЙ ЦЕНТР ГОСУДАРСТВЕННОЙ НАУЧНО-ТЕХНИЧЕСКОЙ ЭКСПЕРТИЗЫ"

критериям оценки)				
Обоснованность запрашиваемого финансирования		The total funding requested by the clients is justified for the project of this magnitude. Based on the main quantitative parameters of the application (i.e., the number of researchers, the amount of materials and equipment to be purchased, the number of business trips, etc.), the amount of funding requested by the applicant corresponds to the significance of the project and the actual amount of funds needed to achieve its goal and expected results. There is a need for major adjustments in the requested funding in particular, the applicants should consider increasing the sample size of the study while lowering cost of labor in order to help strengthen the achievement of the study objectives.	The coherence between the proposed activities cost, also in terms of researcher’s costs, and the justification of the proposed budget is acceptable and no adjustments are required.	Финансирование позволит обеспечить все необходимые ресурсы для проведения исследования, включая оплату труда, командировки, приобретение оборудования и публикацию результатов. Это обеспечит успешное выполнение проекта и достижение его целей.
Соответствие приоритетному направлению		The application corresponds to the priority area and specialized science area under: 1.2 Science of life and health. 1.3. The name of the specialized scientific field in which the application is submitted is: Advanced research in medicine and public health; Area of research in accordance with the Classifier of scientific fields: 1.4 Medicine and health. All components of the proposal are geared towards the evaluation of volume changes induced by hemodialysis on ventriculo-arterial coupling (VAC) computed from echocardiography among patients with ESRD. The project will expand existing knowledge in the fields of cardiology and nephrology. Thus, the proposal fits the priority area and specialized scientific area it was submitted.	The main aim of this project concerns a crucial argument for public health and falls within a high priority medical area. Criteria for inclusion/exclusion of patients, and clinical endpoints, are well described. The proposal is of interest and it well fits within this research field.	1. Медицинская и социальная значимость: Исследование направлено на улучшение качества жизни пациентов с хпн, что является одной из ключевых задач здравоохранения. 2. Инновационные методики: Использование передовых методов неинвазивной диагностики, таких как спекл-трекинг эхокардиография и анализ кривых соотношения давление-объем, способствует внедрению инновационных подходов в клиническую практику. 3. Междисциплинарный подход: Проект объединяет знания и методы из кардиологии, нефрологии, биоинженерии и медицинской визуализации, способствуя развитию комплексного подхода к исследованию и лечению заболеваний. 4. Научная новизна:

[illegible]ОЕ ЗАКЛЮЧЕНИЕ
АО "НАЦИОНАЛЬНЫЙ ЦЕНТР ГОСУДАРСТВЕННОЙ
НАУЧНО-ТЕХНИЧЕСКОЙ ЭКСПЕРТИЗЫ"

				Полученные данные будут способствовать углублению понимания механики сердца и артериальной системы у пациентов на гемодиализе, что позволит разработать новые клинические рекомендации и улучшить прогноз для этой группы пациентов.
Сильные стороны		• The study will help in the evaluation of volume changes induced by hemodialysis on ventriculo-arterial coupling (VAC) computed from echocardiography among patients with ESRD. • The study has clear objectives, well developed research plan, clear inclusion and exclusion criteria, and well written data collection and statistical analysis plan. • The study provided opportunity for the identification of potential new prognostic factors among ESRD patients undertaking hemodialysis.	The project may contribute to characterize a noninvasive assessment of the cardiovascular efficiency and the changes induced by volume changes induced by hemodialysis in patients presenting with end-stage renal disease, also showing a great potential application in personalizing the optimal dialysis modalities and identifying those patients who are at higher risk of left ventricular remodeling and future cardiovascular events.	Высококвалифицированная команда: Включает опытных кардиологов, биоинженеров и специалистов по эхокардиографии с международным признанием, что обеспечивает высокий уровень исследований. Инновационные методики: Использование передовых методов неинвазивной диагностики, таких как спекл-трекинг эхокардиография и анализ кривых соотношения давление-объем (PV loop), обеспечивает точность и надежность результатов. Междисциплинарный подход: Объединение знаний из кардиологии, нефрологии, биоинженерии и медицинской визуализации способствует комплексному пониманию исследуемой проблемы. Международное сотрудничество: Сотрудничество с ведущими международными экспертами и участие в международных конференциях обеспечивает доступ к передовым знаниям и технологиям. Практическая значимость: Результаты исследования позволят улучшить методы диагностики и лечения пациентов с терминальной стадией почечной недостаточности, что повысит качество их жизни и снизит риск сердечно-сосудистых осложнений. Эти сильные стороны

ЭКСПЕРТНОЕ ЗАКЛЮЧЕНИЕ
АО "НАЦИОНАЛЬНЫЙ ЦЕНТР ГОСУДАРСТВЕННОЙ НАУЧНО-ТЕХНИЧЕСКОЙ ЭКСПЕРТИЗЫ"

				обеспечивают высокую вероятность успешного выполнения проекта и достижения его целей.
Слабые стороны		• The relatively low sample size of the study compared to the total funding requested. This is likely to affect the quality of research output and journal of publication. Therefore, I recommend the sample size of the study be increased. • The study did not provide adequate opportunity for the training of undergraduate and postgraduate students. There should be a clear plan for training of young researchers as part of the project	The study does not include details regarding the baseline characteristics and medications of recruited patients, including a stratification based on the main cause of renal failure. Moreover, there is no clear indication about issues related to the prevention of plagiarism, falsification, and fabrication of data, false co-authorship, and the assignment of results.	Зависимость от технологий: Проект требует использования специализированного программного обеспечения и оборудования, что может создать риски в случае технических сбоев или задержек в поставках. Высокие финансовые затраты: Значительное финансирование необходимо для оплаты труда, командировок, приобретения оборудования и публикации результатов. Недостаток финансирования может замедлить прогресс или повлиять на качество исследования. Комплексность координации: Включение большого числа участников из различных учреждений и стран требует сложной координации и может привести к организационным трудностям. Ограниченность выборки: Исследование проводится на специфической группе пациентов (терминальная стадия почечной недостаточности), что может ограничить обобщаемость результатов на более широкую популяцию. Этические и регуляторные барьеры: Проект включает инвазивные процедуры и требует строгого соблюдения этических норм и регуляторных требований, что может усложнить процесс получения разрешений и проведения исследований. Эти слабые стороны требуют тщательного планирования и управления для минимизации рисков и успешного выполнения проекта.

ЭКСПЕРТНОЕ ЗАКЛЮЧЕНИЕ
АО "НАЦИОНАЛЬНЫЙ ЦЕНТР ГОСУДАРСТВЕННОЙ НАУЧНО-ТЕХНИЧЕСКОЙ ЭКСПЕРТИЗЫ"

АО "Национальный центр государственной научно-технической экспертизы" ______________________

Мемлекеттік
ғылыми-техникалық
сараптама

Государственная
научно-техническая
экспертиза
