# Supplementary material for: Intradialytic Changes and Prognostic Value of Ventriculo-Arterial Coupling in Patients With End-Stage Renal Disease: Protocol for an Observational Prospective Trial
Source: JMIR Res Protoc. 2025 Jun 23;14:e71948. doi: 10.2196/71948 (PMC12235198; doi:10.2196/71948)
Supplement: Multimedia Appendix 6 [file resprot_v14i1e71948_app6.docx]

**
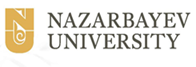
**

**Informed Consent Form**

**Introduction**

You have been invited to participate in a research study entitled ***“Ventriculo-arterial coupling in patients with end-stage renal disease on hemodialysis: Intra-dialytic changes and prognostic value”*** conducted by Alessandro Salustri (Principal Investigator, P.I.), Professor of Medicine at the Nazarbayev University in Astana, Kazakhstan.

**Procedures.**

An echocardiogram (cardiac ultrasound) will be performed by a sonographer before and after your hemodialysis session. A probe will be placed on your chest while you are comfortably lying on a bed to acquire the images of your heart. The echocardiogram has no harm, and the duration of the test is about 15 minutes.

**Risks**

Echocardiography is a noninvasive procedure with no known risks. The sonographer will have minimal contact with you, and the procedure will be clearly explained beforehand. No additional stress or discomfort is expected.

**Benefits**

The results of this study will allow a better understanding of the effect of hemodialysis on the cardiovascular system with the potential of identifying those patients who are at high risk of future cardiovascular events.

**Compensation**

No tangible compensation will be given. A copy of the research results will be available at the conclusion of the study.

**Confidentiality & Privacy**

All information collected will be stored on a secure, password-protected computer, with access limited to the principal investigator and research supervisor. Data will be encrypted both during storage and transfer to ensure it remains secure. Multi-factor authentication (MFA) will further protect access. To maintain confidentiality, your personal information will be replaced with a unique code, with the key linking it to you stored separately. Regular secure backups and audits will be conducted to ensure compliance with data protection standards. All data will be anonymized, and every effort will be made to safeguard your privacy. You are free to withdraw from the study at any time, in which case your data will be permanently deleted.

**Voluntary Nature of the Study**

Participation in this study is strictly voluntary, and if agreement to participation is given, it can be withdrawn at any time without prejudice.

**Points of Contact**

You may address any questions or comments arise regarding this project, or a research related injury is received, to the Principal Investigator:

Alessandro Salustri, MD

Professor of Medicine

NUSOM – Astana, Kz

E-mail: [alessandro.salustri@nu.edu.kz](mailto:alessandro.salustri@nu.edu.kz)

Tel.: +77052264843

This study has been reviewed and cleared by the Nazarbayev University Institutional Research Ethics Committee. If you have concerns or questions about your rights as a participant or about the way the study is conducted, you may contact: Nazarbayev University Institutional Research Ethics Committee. E-mail: [resethics@nu.edu.kz](mailto:resethics@nu.edu.kz).

**Statement of Consent**

I, __________________________________________________________________,

- Give my voluntary consent to participate in this study.
- The researchers clearly explained to me the background information and objectives of the study and what my participation in this study involves.
- I understand that my participation in this study is voluntary. I can at any time and without giving any reasons withdraw my consent, and this will not have any negative consequences for myself .
- I understand that the information collected during this study will be treated confidentially.

Signature: _______________________ Date: ___________________

Researcher:
